# Towards precise classification of cancers based on robust gene functional expression profiles

**DOI:** 10.1186/1471-2105-6-58

**Published:** 2005-03-17

**Authors:** Zheng Guo, Tianwen Zhang, Xia Li, Qi Wang, Jianzhen Xu, Hui Yu, Jing Zhu, Haiyun Wang, Chenguang Wang, Eric J Topol, Qing Wang, Shaoqi Rao

**Affiliations:** 1 Department of Computer Science, Harbin Institute of Technology, Harbin 150001, China; 2 Department of Bioinformatics, Harbin Medical University, Harbin 150086, China; 3 School of Biological Science and Technology, Tongji University, Shanghai, 200092, China; 4 Department of Molecular Cardiology and Department of Cardiovascular Medicine, the Cleveland Clinic Foundation, 9500 Euclid Avenue, Cleveland, Ohio 44195, USA

## Abstract

**Background:**

Development of robust and efficient methods for analyzing and interpreting high dimension gene expression profiles continues to be a focus in computational biology. The accumulated experiment evidence supports the assumption that genes express and perform their functions in modular fashions in cells. Therefore, there is an open space for development of the timely and relevant computational algorithms that use robust functional expression profiles towards precise classification of complex human diseases at the modular level.

**Results:**

Inspired by the insight that genes act as a module to carry out a highly integrated cellular function, we thus define a low dimension functional expression profile for data reduction. After annotating each individual gene to functional categories defined in a proper gene function classification system such as Gene Ontology applied in this study, we identify those functional categories enriched with differentially expressed genes. For each functional category or functional module, we compute a summary measure (s) for the raw expression values of the annotated genes to capture the overall activity level of the module. In this way, we can treat the gene expressions within a functional module as an integrative data point to replace the multiple values of individual genes. We compare the classification performance of decision trees based on functional expression profiles with the conventional gene expression profiles using four publicly available datasets, which indicates that precise classification of tumour types and improved interpretation can be achieved with the reduced functional expression profiles.

**Conclusion:**

This modular approach is demonstrated to be a powerful alternative approach to analyzing high dimension microarray data and is robust to high measurement noise and intrinsic biological variance inherent in microarray data. Furthermore, efficient integration with current biological knowledge has facilitated the interpretation of the underlying molecular mechanisms for complex human diseases at the modular level.

## Background

Gene expression profile (GEP) has been widely used to address the relationship between disease phenotypes and the cellular expression patterns. Numerous data mining methods have been proposed for precise classification of disease phenotypes (subtypes) using high dimension GEPs [1-5]. Although much progress in applying microarray technology to versatile biological kingdoms has been witnessed in recent time, further advancing its efficiency and power in elucidating complex biological mechanisms would very likely rely on our ability to handle the high dimension genetic information mixed with measurement noises [6,7], intrinsic biological variance [8,9], and a large number of irrelevant genes [10,11]. However, lack of coherence in biological interpretations often occurring in analysis of gene expression profiling can be remedied partially by integrating with a knowledge-mining tool such as Onto-Express developed by Draghici et al. [12,13].

Cellular biology is essentially to study an interacting network of various functional gene modules that coordinately carry out highly integrated cellular functions in somewhat isolated fashions [14-16]. The assumption that genes express and perform their functions in modular fashions in cells has been supported by accumulated multiple lines of evidence from, among others, gene expression and protein-protein interaction studies [17-19]. Inspired by the insight that genes often interplay as a module to realize a highly integrated cellular function, we propose an alternative approach to analyzing the high dimension microarray data by formulating the disease classification problem from a perspective of modularity. In this study, we map genes to their categories in Gene Ontology (GO) [20,21], which provides a unified gene function classification system across genomes. After annotating each individual gene to a GO functional category, we identify gene functional categories enriched with differentially expressed genes. These categories, defined as differentially expressed functional modules, are very likely to be relevant with experimental conditions, or specifically, with the disease type discrimination. For each functional module, we construct a representative functional feature, and then employ a traditional data mining toolbox to train the rule(s) for classifying disease types based on the newly built functional expression profiles (FEPs). Instead of analyzing raw expressions of single genes, we consider the gene expressions within a functional module as an integrative data point to shrink the feature dimension. This modular approach is flexible and also statistically robust to high measurement noise and intrinsic biological variance inherent in microarray data. Furthermore, efficient integration with current biological knowledge support provided in the GO database has facilitated the interpretation of the underlying molecular mechanisms at the modular level. We apply the alternative approach to analyze four publicly available datasets to demonstrate its performance and statistical properties. The results from analysis of two datasets (NCI60 dataset [1] and the lymphoma dataset [2]) are described in the main text. To obtain a robust and convincing comparison of FEP and GEP, we have undertaken analysis of two additional large-scale datasets and have described the detailed results in the supplement [see Additional file 1].

## Results

### Description of the two datasets

NCI60 dataset consists of 9,703 cDNAs whose expression levels were measured in 60 cancer cell lines derived from a variety of tissues and organs. A subset of NCI60 (31 samples of four cancer types) is used in this study, including 8 samples from renal cancer (RE), 7 samples from colon cancer (CO), 8 samples from leukaemia (LE), 8 samples from melanoma (ME), respectively. The same criterion as in [1] is used to identify the differentially expressed genes (i.e., log_2 _(*ratio*) > 2.8 or log_2 _(*ratio*) < -2.8 in at least four cell lines). A total of 1160 genes are filtered. The lymphoma dataset contains the expression profiles of 18,000 cDNAs for 42 samples of the diffuse large B-cell lymphoma (DLBCL), 9 samples from follicular lymphoma (FL), 11 samples from chronic lymphocyte leukaemia (CLL) and 24 samples from the healthy sources (NORMAL) prepared from activated human blood B cells and resting/activated blood T cells, respectively. The 4,026 genes, originally filtered by Alizadeh et al. [2], is used in this study. As suggested by Alizadeh et al. [2], we exclude 9 samples (eight NORMAL samples and one DLBCL sample). Using the criterion "log_2 _(*ratio*) > 1 or log_2 _(*ratio*) < -1 in at least eight cell lines" [2], we identify a total of 705 differentially expressed genes.

### FEP based analysis of NCI60 dataset

Based on NCI60 dataset, 114 differentially expressed GO modules are identified according to a statistical test described in the Methods section and their functional expression profiles, a 114 × 31 matrix, are denoted with FEP114A or FEP114M when arithmetic mean or median is used to summarize the overall activity of a module, respectively. The 114 differentially expressed GO categories are annotated with a total of 617 differentially expressed genes. For comparison, we also perform classification analysis using the expression profiles of the 617 differentially expressed and annotated genes (GEP617) or the 1160 differentially expressed genes (GEP1160).

**Figure 1:**
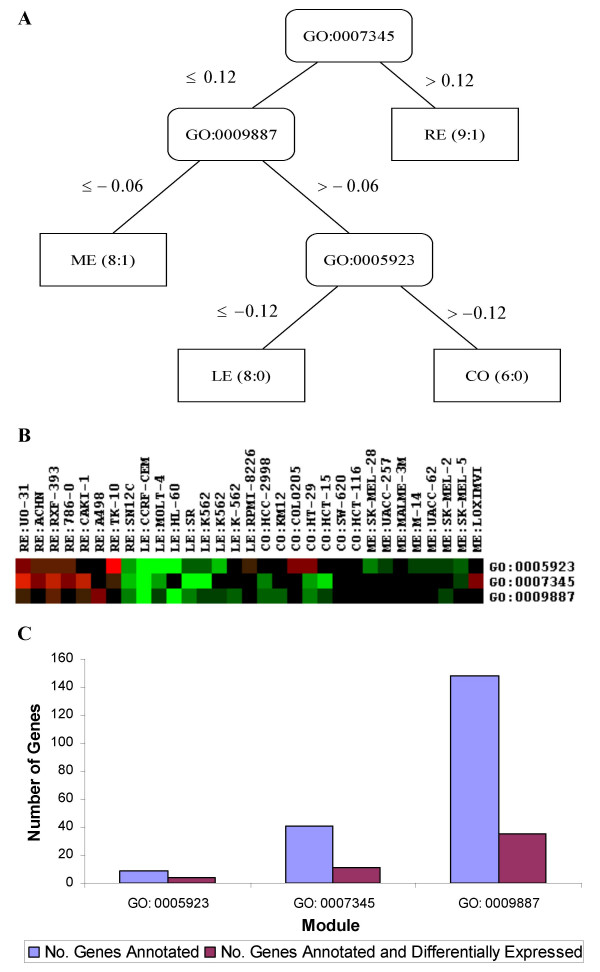
**Training classification rules for four cancer types based on functional expression profiles of 114 modules. A **– Decision tree trained with the NCI60 FEP median measure. The internal nodes of the tree are denoted with the functional modules from Gene Ontology. The leaf nodes give the classification results for the cancer types. The numbers in the leaf nodes are the total number of samples contained over the number of the incorrectly predicted samples. **B **– Functional expression profiles of the three identified modules. For the identified GO modules from decision analysis, their functional expression profiles are demonstrated with a colouring spectrum of their medians. Each GO module corresponds to a row, and the column denotes the functional expression for each cell line. At the top are names of cell lines (renal cancer (RE), colon cancer (CO), leukaemia (LE), melanoma (ME)). Samples with a missing value or the null value are coded with black colour, a positive with red colour and a negative with green colour. **C **– numbers of genes annotated and differentially expressed in the three identified modules.

Recursive partition analysis of the 114 functional features using median as the summary measure identify three significant functional signatures for multiple cancer subtypes. Figure 1A depicts the decision tree trained on the FEP114M. The internal nodes of the tree are denoted with the functional modules from Gene Ontology. The leaf nodes give the classification results for different cancer types: the total number of samples contained over the number of the incorrectly predicted samples. Figure 1B depicts the functional expression profiles of the three modules (GO:0005923, GO:0007345 and GO:0009887). The three modules are annotated with 9, 41 and 148 genes (figure 1C), respectively. We identify 4, 11 and 35 genes that are differentially expressed between four cancer types, respectively. Hypergeometric tests indicate that all the three modules are significantly (or highly significantly, *p*-value < 0.01) enriched with differentially expressed genes, with the probability of observing a more extreme of 0.0150, 0.0322 and 0.0079, respectively.

One advantage for FEP based analysis is to allow us to interpret our findings at the modular level. Based on the trained tree, we observe that RE is distinct from the remaining cancer types and is characterized with the up-regulation of GO:0007345 (termed with embryogenesis and morphogenesis), suggesting that the abnormal up-regulation (possibly over-expression) of the genes that determine embryogenesis and morphogenesis may contribute to development of RE, too. To look for knowledge support, we search G2D database [22,23]. Interestingly, significant association of GO:0007345 with RE has been documented previously. PUBMED searching provides further evidence to support the trained hypothesis. Gene F2R (thrombin receptor), which is differentially expressed between the investigated cancers and is also annotated in the module, is pivotal in proliferation and motility of prostate cancer cells [24], colon cancer cells [25] and breast carcinoma cells [26]. We thus propose that GO:0007345 may be an important functional target for the molecular pathogenesis of RE. Further distinction between the remaining three cancers can be made by looking at the module GO:0009887, which acts for organogenesis and is down-regulated in ME, but is up-regulated in both LE and CO. G2D database indicates that GO:0009887 is indeed significantly associated with both LE and CO. By searching PUBMED, we find that *CYP1B1 *(a member of cytochrome P450 enzyme), a differentially expressed gene annotated in this module, was reported to be associated with high risk for developing several forms of cancers [27], which is again consistent with our finding. The third module, GO:0005923, contains a cluster of genes for tight junction and is identified for distinguishing between caners LE and CO. Its association with LE has been documented in G2D. In addition, experiment studies agree with our finding that three of the four differentially expressed genes (*CLDN1*, *CLDN4 *and *CLDN5*) annotated in the module are members of the claudin family, which were demonstrated to be related to the invasiveness and metastatic phenotype of pancreatic and colorectal cancers [28,29]. In short, the above biological knowledge mining supports our analysis. Intuitively, the functional expression patterns, as demonstrated in figure 1B, are clearly distinguishable between the four cancers. RE samples have the highest expressions in all three modules and ME samples have the lowest. Nevertheless, two outliers (RE:SN12C and ME:L0XIMVT) have marked deviations from their respective groups and thus not surprisingly they have been misclassified.

**Figure 2:**
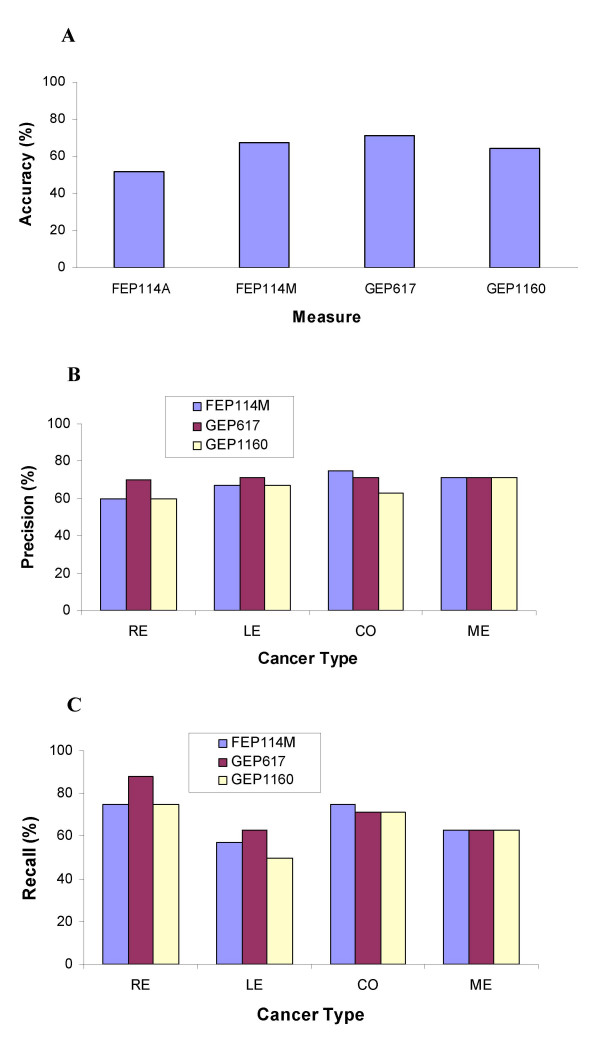
Comparison of different gene expression measures for classification of cancer types in terms of accuracy (A), precision (B) and recall (C).

To provide an unbiased evaluation on the utility of the trained three modules for multi-class cancer diagnosis, we perform a five-fold cross validation procedure, as described in the Methods section, to verify the trained classifier in terms of accuracy, precision and recall. As shown in figure 2A, the classification accuracies for four gene expression measures (FEP114A, FEP114M, GEP617 and GEP1160) are 51.6%, 67.7%, 71.0% and 64.5%, respectively. Comparing the two summary measures, median (FEP114M) generally perform better than mean (FEP114A), evaluated in terms of the three criteria. Using the low dimension function profile and median measure, we have achieved comparable results to those using the high dimension gene expression profiles (GEP617 and GEP1160). However, this application implicates that there is a space for further improvement in multi-class cancer diagnosis using tumour gene expression signatures or functional signatures, perhaps by combining with the other contributed clinical risk factors and histopathological information, to some extent which has reflected the complex nature of cancers.

### FEP based analysis of the lymphoma dataset

For the lymphoma dataset, 44 differentially expressed GO modules are identified and their functional expression profiles make up a 44 × 77 matrix, called FEP44A or FEP44M when arithmetic mean or median is used to be the summary measure, respectively. The 44 differentially expressed GO modules are annotated with a total of 383 differentially expressed genes. Again for comparison, we also perform classification analysis using the raw expression profiles of the 383 differentially expressed and annotated genes (GEP383) or the 705 differentially expressed genes (GEP705).

**Figure 3:**
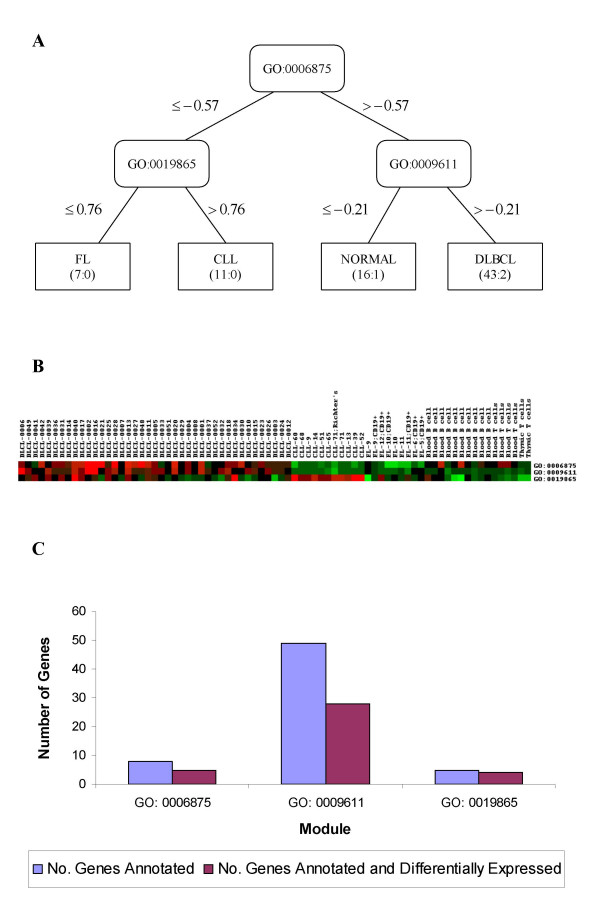
**Training classification rules for lymphoma subtypes based on functional expression profiles of 44 GO modules. A **– Decision tree trained with the lymphoma FEP median measure. The internal nodes of the tree are denoted with the functional modules from Gene Ontology. The leaf nodes give the classification results for the lymphoma subtypes. The numbers in the leaf nodes are the total number of samples contained over the number of the incorrectly predicted samples. **B **– Functional expression profiles of the three identified modules. For the identified GO modules from decision analysis, their functional expression profiles are demonstrated with a colouring spectrum of their medians. Each GO module corresponds to a row, and the column denotes the functional expression for each cell line. At the top are names of cell lines (diffuse large B-cell lymphoma (DLBCL), follicular lymphoma (FL), chronic lymphocyte leukaemia (CLL), and the healthy sources (NORMAL)). Samples with a missing value or the null value are coded with black colour, a positive with red colour and a negative with green colour. **C **– Numbers of genes annotated and differentially expressed in the three identified modules.

By a coincidence, we also identify three functional modules for distinguishing lymphoma subtypes. Figure 3A displays the decision tree trained on FEP44M of the lymphoma dataset. Figure 3B gives the expression patterns of the three functional modules (GO:0006875, GO:0009611 and GO:0019865) for 77 tissue samples. Over half of annotated genes in all the modules are differentially expressed between the tissue samples (figure 3C), i.e., 5, 28 and 4 out of 8, 49 and 5 genes measured in the three modules, respectively. Hypergeometric tests indicate that all the three modules are significantly (or highly significantly, *p*-value < 0.01) enriched with differentially expressed genes, with the probability of observing a more extreme of 0.0396, 0.0008 and 0.0205, respectively.

**Figure 4:**
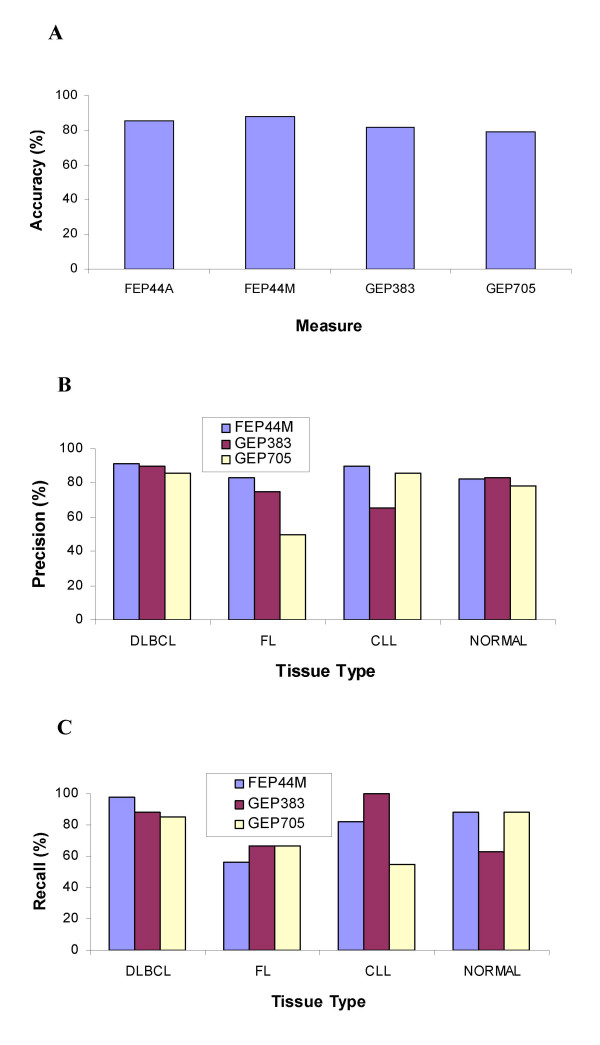
Comparison of different gene expression measures for classification of lymphoma tissues in terms of accuracy (A), precision (B) and recall (C).

Because G2D database lacks the data for DLBCL and FL, we resort to PUBMED for knowledge support. The first identified module, GO:0006875 (metal ion homeostasis) is a parental category of GO:0006874 (calcium ion homeostasis). Three genes (*Hs.241392*, *Hs.73817 *and *Hs.237356*) in the small size module are differentially expressed between the lymphoma tissue types. Anghileri et al. [30] showed that calcium-overload can lead to proliferation and neoplastic transformation of lymphocytes in mice and suggested the involvement of the calcium homeostasis change in lymphoma induction. At the second layer of the trained tree, GO:0009611 (response to wounding) distinguishes DLBCL (up-regulated, clearly visible in figure 3B) from normal samples. One differentially expressed gene annotated in this GO module, *BCL6 *(zinc finger protein 51), was found to be frequently translocated and hypermutated in diffuse large-cell lymphoma (DLBCL), and it thus may be involved in the pathogenesis of DLBCL [31]. The functional module labelled immunoglobulin binding (GO:0019865) may be an important modular marker for separating the two lymphoma subtypes (FL and CLL). One differentially expressed gene annotated in this GO module, *CD23 *(Fc fragment of IgE, low affinity II), was identified to be associated with chronic lymphocytic leukaemia (CLL) [32], which is again consistent with our data. Median measure FEP44M achieves the highest accuracy (88.31%) for classification of lymphoma tissue types (figure 4A). Again, as we expected, median perform better than mean (FEP44A) in terms of accuracy, precision and recall. Of special note, FEP44M attains a nearly perfect precision or recall rate (98%) to distinguish DLBCL from others (figures 4B and 4C), implicating its utility to clinically isolate this particular lymphoma using the identified modular signatures.

We present in Additional file 1 the detailed numeric results for further comparison of different gene expression measures using four datasets (plus two additional large-scale datasets). In all the four datasets, FEPs have achieved comparable or better classification performance than those GEPs do. In short, we have provided convincing evidence to support FEP as a robust gene expression measure, as a useful summary index for efficient data reduction and as a way towards precise classification of biological phenotypes at the modular levels.

## Discussion

With the rapid accumulation of gene functional knowledge, GO functional modules have been widely applied in inferring the unknown functions of genes based on their expression profiles (e.g. [33-35]), but there is an open space for development of the timely and relevant computational algorithms that use robust functional expression profiles towards precise classification of complex human diseases at the modular level. In this paper, we have presented an alternative approach to analyzing microarray data. The central idea is to transform the gene expressions to modular level to achieve both robustness and precise classification with better biological interpretation. We first map genes onto their functional modules according to GO hierarchy, and then consider the newly built modules as the features for learning. Because the modules are evaluated with a summary measure(s), its variance is considerably reduced. For this reason, function expression profiling is robust to measurement or biological noises, outliers and distribution assumptions underlying some approaches.

Recent time has witnessed the attempts to study human diseases at the modular levels. Hanczar et al. [36] grouped the whole set of genes with *k*-means clustering of the averaged expression values in each cluster and then trained a SVM classifier based on these integrated values. Huang et al. [37,38] chose a Bayesian formalism of singular value decompositions (SVD) coupled with binary regression and stochastic regularization. Our approach differs from these methods in at least three aspects. First, we construct a module based on the well established GO categories in order to achieve better biological interpretation. Second, we identify statistically significant modules enriched with differentially expressed genes to avoid inclusion of some noise modules. Third, we can easily procure biological knowledge (e.g., GO in this study) because of the very nature of the proposed methodology.

Traditional methods for reducing dimension of gene expression profiles are feature selection [39], for examples, wrappers, filters and embedded algorithms. However, if only an optimal gene subset is extracted, many genes of the same (or similar) function(s) would be excluded due to redundancy. We have thus proposed an ensemble approach to mining disease-relevant genes by constructing a gene forest [40,41]. Alternatively, one may consider analyzing the gene expression profiles at the modular level to avoid unnecessary loss of important information. The proposed modular approach can achieve both goals simultaneously: reducing the dimension of microarray data by transforming the single gene expressions into modular expressions and improving the interpretability on the data mining results. The trained functional modules can be presented graphically and are easily understood by biologists. In fact, a trained tree implicates a decision rule(s) that determine the interactions of modules and can be used to elucidate the complex cellular processes that lead to distinct biological types. Our approach could be regarded as a way of identifying disease-relevant functional modules (selected by decision trees) guided by precise classification of cancers. Compared with many tools (e.g. Onto-Express, FatiGO, GoMiner [42] and GOAL [43]) developed mainly for gene function annotation using the data acquired from microarrays or other high-throughput techniques, our approach focuses on identification of the modules of high disease discriminating power, thus implicating stronger evidence of their relevancy with the studied disease. However, caution should be taken in interpretation of the module selection for refinement of the biological phenotypes investigated, especially when normal controls are not included. In this case, the modules relevant to disease subtypes should be considered as important molecular signatures which may also be the disease-causing modules.

In the study, genes are annotated to the modular terms in GO as granted. Nevertheless, the classification system with modules hierarchically structured is neither the most efficient nor the optimal for pursuing specific biological tasks, for example, classifying cancer types using modular signatures. In the context of microarray experiments, a large number of cDNA sequences often remain not being annotated by GO because of either their unknown functions or ambiguous annotations. To extract maximal information from microarray data, one may consider performing computational function assignments of gene products using the strategy proposed by Vinayagam et al. [44]. Therefore, further investigations on an alternative classification system(s) or an extension of the GO system and choices of more efficient indices for functional expression profiling are warranted.

## Conclusion

In summary, we have proposed an alternative approach to analyzing gene expression profiles at the modular levels, where the functional expression profiles replace the traditional gene expression profiles. We have applied the alternative approach to four large-scale microarray datasets, and have achieved comparable or better classification performance by using the functional expression profiles. It should be recognized that median or other modular measures are generally robust to noises because they are less sensitive to any single individual gene expression value. However, for the same reason, they are conservative in using full information of microarray experiments, so it cannot be vouched that FEP always has better performance than GEP does. Despite this fact, the improved biological interpretability and the advantages of robustness to measurement noise and intrinsic biological variance of gene expression data would promote its application in biomedical research.

## Methods

### Gene annotation and definition of the differentially expressed functional modules

In GO database, gene functional categories are tagged with functional terms and organized in three directed acyclic graphs where the root nodes are tagged with "biological process" (BP), "molecular function" (MF) and "cellular component" (CC), respectively. There are two kinds of relationships between a child category and its parent categories in GO: 'Is-a', where the child category is an instance of its parents, and 'Part-of', where the child category is a part of its parents. Up to the present, GO contains a total of over 17,000 categories (or called modules), with 8625 categories in the BP ontology, 1407 categories in the CC ontology, and 7336 categories in the MF ontology. All the information in GO can be downloaded in a relational database file format to local computers. With the existing gene function knowledge, known genes can be annotated to certain GO categories corresponding to their most specific function(s). As implied by the ontology structure, one gene annotated to a category is also within the ancestor categories on the same path.

During the annotation step, a gene can be annotated with multiple GO categories. Not all of these categories, however, are to be used in this study. Only the categories that contain significantly larger number of differentially expressed genes than by random are kept for the following analysis. As Khatri et al. [45] and Al-Shahrour at al. [46,47] did, we perform a statistical test to decide whether a GO category is significantly enriched with differentially expressed genes that are aroused (induced or repressed) by the experiment conditions. Suppose that a total of *N *genes (set A) for the analyzed data are annotated in GO in which a set of *C *genes (set B) are differentially expressed. For a given GO category, a gene is either in the category or not in the category. Suppose further that *n *genes out of set A and *k *genes out of set B are in the category. If the *k *differentially expressed genes are effectively a random sample uniformly selected from set B, the expected value of *k *is (*n*/N) *C*. As a gene can be selected only once, this is the sampling without replacement and can therefore be appropriately modelled by a hypergeometric distribution [45]. The probability of observing at least *k *differentially expressed genes in the GO category of *n *genes can be computed as follows:



The *p*-value calculated above corresponds to a one-sided test and a smaller *p*-value relates to a higher likelihood of a GO category's enrichment with differentially expressed genes. Only the categories that contain significantly (*p *≤ 0.05) larger number of differentially expressed genes than that by random are kept for the following analysis. In this study, to avoid the possible loss of the true positives, we do not perform a multiple-tests correction for multiple GO categories evaluated. Therefore, the *p*-value quoted should be considered as a heuristic measure, useful as an indicator of roughly rating of the relative enrichment of differentially expressed genes for each GO category. We remove a redundant category if all the genes annotated to a category are also annotated to one of its child categories. In this case, we retain one of the child categories because its function(s) is more specifically defined. In the following text, we refer to a GO category significantly enriched with differentially expressed genes as a 'gene functional module', or a 'module' for short.

### Construction of the functional expression profiles

After extracting the differentially expressed functional modules, we compute two summary measures: arithmetic mean and median (the 50% quantile) of all the gene expression values in each module to capture the activity of the module. The modular measure(s) can have multiple sources of variations including systematic experiment variation, treatment effects, chip variation and biological variation [6-9]. The distributions for individual (raw) gene expression are usually not known in prior and could be contaminated with outliers and possibly distorted due to heteroscedasticity [48]. Therefore, mean or median measure for the modular activity can be good remedy statistics for the location parameter. When the data are Gaussian or symmetrically distributed, sample mean has a higher statistical efficiency compared to sample median. If there are outliers, however, sample median is a robust measure for the modular activity [49].

### Evaluation of the functional profiles based on a decision tree

Based on the functional expression profiles computed with the two summary measures, we can now apply a proper classification algorithm as do traditionally for the individual measures of gene expressions. In this study, we chose a decision tree model (e.g., C4.5 [50,51]) to train the classification rule. Since there often are only limited numbers of instances in microarray experiments, we adopt a *k*-fold (*k *= 5 in this study) cross-validation procedure. In the *k*-fold cross-validation, we divide the data into *k *subsets of approximately equal size. We train the classifier on the *k*-1 subset and use the remaining subset to test the performance of the classifiers. The performance for each classifier is evaluated in terms of three measures: accuracy, precision and recall rate, which are defined as follows:



where *TP*, *TN*, *FP *and *FN *denote true positive, true negative, false positive and false negative, respectively. Each sample in the test set can be categorized in one of the four outcomes. True positives are class members according to both the classifier and sample label (disease type). True negatives are non-members according to both. False positives are samples that the classifier places within the given class, but sample labels are non-members. False negatives are samples that the classifier places outside the class, but sample labels are members. Accuracy is a percentage quantity for the number of times that the classifier is correct in its classification and it conveys the right intuition when the positive and negative populations are roughly equal in size. Precision is the percentage of times that the classifier is correct in its classification of positive samples. Recall is the percentage of known positive samples that the classifier would classify as being positive.

### Biological knowledge support

We apply G2D (Candidate Genes to Inherited Diseases) database [22,23] to associate a phenotype (disease) to a GO module trained using a decision tree. G2D database is built by text-mining approach. First, the number of papers in MEDLINE that contain a MeSH-C term (describing a phenotypic feature) and a MeSH-D term (describing a chemical object) are counted, and then the corresponding phenotypic term and the chemical term are judged as associated if they occur together in many abstracts. Second, a chemical term is judged as associated to a GO term corresponding to a functional module appearing on the decision tree if they appear to be related by many sequences from RefSeq. Third, if a phenotypic term is associated to a chemical term that has a functional counterpart, then the phenotypic term is associated to the functional term. We search PUBMED manually to get further supporting evidence. If one or several differentially expressed genes, which are annotated to one functional module in the decision tree, are suggested by existing literature to be functionally related to one disease type, the investigated functional module is then deemed to be functionally relevant to the disease type.

## List of abbreviations used

Gene expression profile (GEP), function expression profile (FEP), Gene Ontology (GO), renal cancer (RE), colon cancer (CO), leukaemia (LE), melanoma (ME), diffuse large B-cell lymphoma (DLBCL), follicular lymphoma (FL), chronic lymphocyte leukaemia (CLL), healthy sources (NORMAL), arithmetic mean (A), median (M), Candidate Genes to Inherited Diseases (G2D), biological process (BP), molecular function (MF), cellular component (CC).

## Authors' contributions

This study was undertaken by a collaborative team of four institutes as indicated, led by ZG, TZ, XL and SR, who also conceived of the proposal of the study and drafted the manuscript. JX, QW (Qi Wang) and HY participated in writing the computing codes and applied the data mining strategy to the field datasets. JZ, HW, CW, EJT and QW (Qing Wang) implemented the search for biological knowledge support and provided constructive advice for the biological interpretation of the results. All authors participated in reading, approving and revising the manuscript.

## Supplementary Material

Additional File 1 Further comparison of different gene expression measures for classification of biological phenotypes using four large-scale datasets Click here for file
