# Supplementary material for: Towards precise classification of cancers based on robust gene functional expression profiles
Source: BMC Bioinformatics. 2005 Mar 17;6:58. doi: 10.1186/1471-2105-6-58 (PMC1274255; doi:10.1186/1471-2105-6-58)
Supplement: Additional File 1 — Further comparison of different gene expression measures for classification of biological phenotypes using four large-scale datasets [file 1471-2105-6-58-S1.doc]

# Further comparison of different gene expression measures for classification of biological phenotypes using four large-scale datasets

― Additional supporting analyses for the article: Guo Z, Zhang T, Li X, Wang Q (Qi), Xu J, Yu H, Zhu J, Wang H, Wang C, Topol EJ, Wang Q (Qing), Rao S: **Towards precise classification of cancers based on robust gene functional expression profiles**. *BMC Bioinformatics* 2005, **6**.

# Materials

## In the additional report for the further detailed comparison of different gene expression measures for classification of biological phenotypes, we will present and discuss the results from analysis of four large-scale datasets. The NCI60 dataset and the lymphoma dataset have been already described in the main article.

## Description of the leukaemia microarray data

The leukaemia data are publicly available at http://www.stjuderesearch.org/data/ALL1. The data consist of gene expression profiles of 327 ALL (acute lymphoblastic leukaemia) samples [1]. The profiles were obtained by hybridisation on the Affymetrix U95A GeneChip containing probes for 12,558 genes. The samples were labelled with six known acute lymphoblastic leukaemia subtypes. We select the two subtypes with the largest sample sizes, which contains gene expression levels of 143 patients either suffering from TEL-AML1 (B lineage leukaemia that contain t(12;21), 79 cases) or HH (hyperdiploid hyperdip > 50, 64 cases). Again, we work with the pre-processed data as implemented in Dudoit et al. [2]. The final data have the expression values of 8677 genes, of which a total of 1098 differentially expressed genes were identified by Student’s t-test (at significance level = 0.001) based on comparing the differences in log-intensities (Affymetrix microarrays) between two disease classes relative to the variation expected in the mean differences.

## Description of the microarray data of prostate cancer

The prostate cancer data, publicly available at <http://genome-www5.stanford.edu/>, consist of gene expression profiles of 62 primary prostate tumours and 41 normal prostate specimens. The cDNA microarrays contain 26,260 genes (UniGene clusters) [3]. We work with the pre-processed subset of the data as described in Dudoit et al. [2]. The final data have the transcriptional values of 11,763 genes, of which a total of 1938 differentially expressed genes were identified by Student’s t-test (at significance level = 0.001) based on comparing the differences in mean log-ratios (cDNA microarrays) between two phenotypic classes relative to the variation expected in the mean differences.

# Results and Discussions

Based on the datasets for leukaemia and prostate cancer, we identify 24 and 31 differentially expressed GO modules, respectively, according to the statistical test described in the Methods section of the main text. We only use the median summary measure (FEP) to capture the overall transcriptional activity of a module. For each dataset, we compare the FEP classification performance with that of using the expression profiles of either the differentially expressed genes (GEP1) or the raw genes (GEP2). We present the numerical results in Table S1.

By recursive-partition based tree analysis of the leukaemia subtypes, the FEP achieves an averaged classification accuracy of 93% (Table S1), derived from 5-fold cross-validation resampling replicates. We identify two significant molecular modules (GO:0015629, actin cytoskeleton; and GO:0007003, telomere binding). The two GO modules annotate 10 and 2 differentially expressed genes, respectively. Hypergeometric tests indicate that their enrichments are statistically significant (*p*-value < 0.05), with the probability of observing a more extreme of 0.0415 and 0.0429, respectively. Using only one module (GO:0015629) and the cutoff of 11.98, we are already able to separate the two leukaemia subtypes well, with a high accuracy (93% when using the best trained tree), suggesting that the module (actin cytoskeleton) contribute substantially to the molecular differentiation of the underlying pathways for the two leukaemia subtypes. In fact, a recent proteomic research suggested the possible role of tubulin and/or actin cytoskeletons in the vinca alkaloid response and resistance in acute lymphoblastic leukaemia [4].

An averaged classification accuracy of 84% for prostate cancer samples is obtained from the FEP-based analysis of multiple 5-fold cross-validation generated replicates (Table S1). We also identify two relevant molecular modules (GO:0007517, muscle development; and GO:0007566, embryo implantation). The two nodes (modules) appearing in the trained trees have 24 and 3 of the differentially expressed genes, respectively (with the enrichment *p*-values of 0.0007 and 0.0187, respectively). In the best trained tree, the first module (GO:0007517) with the cutoff of 0.52 achieves an accuracy of 90% for classification of the cancer tissue and the health sources. According to Horsfall et al.’s study [5], the age-related increases in prostatic smooth muscle cell size and content of perinuclear organelles in the guinea pig can lead to re-activation of cellular synthetic activity. The similarity in some features of the prostatic smooth muscle stroma between aging men and guinea pigs may implicate common pathophysiologic processes. Therefore, it is not surprising that we observe its high discriminating power of the muscle-associated function module (GO:007517).

# Conclusions

In all four datasets, the median FEP has provided comparable (essentially equivalent) or better classification performance than the GEP1 does. However, compared with the data reduction strategies of either use of a functional modular measure or use of the filtered differentially expressed genes, the direct application of the raw gene expression data (GEP2 in this study), from both views of theory and practice, is not a recommended data analysis strategy. We believe that testing on the additional datasets has proved the robust value of the proposed modular approach towards elucidating underlying biological complexities at the higher levels.

# References

1. Yeoh EJ, Ross ME, Shurtleff SA, Williams WK, Patel D, Mahfouz R, Behm FG, Raimondi SC, Relling MV, Patel A, Cheng C, Campana D, Wilkins D, Zhou X, Li J, Liu H, Pui CH, Evans WE, Naeve C, Wong L, Downing JR: **Classification, subtype discovery, and prediction of outcome in pediatric acute lymphoblastic leukemia by gene expression profiling**. *Cancer Cell* 2002, **1**(2):133-143.

2. Dudoit S, Fridlyand J, Speed T: **Comparison of discrimination methods for the classification of tumours by using gene expression data. , 97(457), 77-87.** *J Am Stat Assoc* 2000, **97**:77-87.

3. Lapointe J, Li C, Higgins JP, van de Rijn M, Bair E, Montgomery K, Ferrari M, Egevad L, Rayford W, Bergerheim U, Ekman P, DeMarzo AM, Tibshirani R, Botstein D, Brown PO, Brooks JD, Pollack JR: **Gene expression profiling identifies clinically relevant subtypes of prostate cancer**. *Proc Natl Acad Sci U S A* 2004, **101**(3):811-816.

4. Verrills NM, Walsh BJ, Cobon GS, Hains PG, Kavallaris M: **Proteome analysis of vinca alkaloid response and resistance in acute lymphoblastic leukemia reveals novel cytoskeletal alterations**. *J Biol Chem* 2003, **278**(46):45082-45093.

5. Horsfall DJ, Mayne K, Ricciardelli C, Rao M, Skinner JM, Henderson DW, Marshall VR, Tilley WD: **Age-related changes in guinea pig prostatic stroma**. *Lab Invest* 1994, **70**(5):753-763.

**Table S1:** Comparison of different gene expression measures for classification of biological phenotypes in terms of rates (%) of accuracy, precision and recall.

| Phenotype | Dataset | Accuracy | | |  | Precision | | |  | Recall | | |
| --- | --- | --- | --- | --- | --- | --- | --- | --- | --- | --- | --- | --- |
|  |  | FEP | GEP1 | GEP2 |  | FEP | GEP1 | GEP2 |  | FEP | GEP1 | GEP2 |
|  |  |  |  |  |  |  |  |  |  |  |  |  |
| RE | NCI60 |  |  |  |  | 60 | 70 | 60 |  | 75 | 88 | 75 |
| LE | NCI60 |  |  |  |  | 67 | 71 | 67 |  | 57 | 63 | 50 |
| CO | NCI60 |  |  |  |  | 75 | 71 | 63 |  | 75 | 71 | 71 |
| ME | NCI60 |  |  |  |  | 71 | 71 | 71 |  | 63 | 63 | 63 |
| 4 cancers | NCI60 | 68 | 71 | 65 |  |  |  |  |  |  |  |  |
|  |  |  |  |  |  |  |  |  |  |  |  |  |
| DLBCL | Lymphoma |  |  |  |  | 91 | 90 | 86 |  | 98 | 88 | 85 |
| FL | Lymphoma |  |  |  |  | 83 | 75 | 50 |  | 56 | 67 | 67 |
| CLL | Lymphoma |  |  |  |  | 90 | 65 | 86 |  | 82 | 100 | 55 |
| Normal | Lymphoma |  |  |  |  | 82 | 83 | 78 |  | 88 | 63 | 88 |
| 4 types | Lymphoma | 88 | 82 | 79 |  |  |  |  |  |  |  |  |
|  |  |  |  |  |  |  |  |  |  |  |  |  |
| HH | Leukaemia |  |  |  |  | 89 | 93 | 90 |  | 93 | 86 | 90 |
| TEL | Leukaemia |  |  |  |  | 95 | 90 | 92 |  | 93 | 95 | 92 |
| 2 types | Leukaemia | 93 | 91 | 92 |  |  |  |  |  |  |  |  |
|  |  |  |  |  |  |  |  |  |  |  |  |  |
| Prostate | Prostate |  |  |  |  | 84 | 82 | 81 |  | 78 | 86 | 84 |
| Normal | Prostate |  |  |  |  | 81 | 88 | 91 |  | 89 | 88 | 87 |
| 2 types | Prostate | 84 | 86 | 86 |  |  |  |  |  |  |  |  |

Abbreviations: FEP, median-based functional gene expression profiles; GEP1, GEP2, individual expression profiles of differentially expressed genes and the raw genes, respectively; RE, renal cancer; LE, leukaemia; CO, colon cancer; ME, melanoma; DLBCL, diffuse large B-cell lymphoma; FL, follicular lymphoma; CLL, chronic lymphocyte leukaemia; HH, hyperdiploid hyperdip >50; TEL (TEL-AML1), B lineage leukaemia that contain t(12;21); Prostate, prostate cancer; and Normal, the healthy control.
